# What Do Resource-Oriented Approaches Mean to General Practitioners and How Can They Be Facilitated in Primary Care? A Qualitative Study

**DOI:** 10.1155/2013/187641

**Published:** 2013-08-06

**Authors:** Franziska Prüfer, Stefanie Joos, Antje Miksch

**Affiliations:** ^1^Department of General Practice and Health Services Research, University Hospital Heidelberg, Voßstraße 2, Geb.37, 69115 Heidelberg, Germany; ^2^Competence Centre for General Practice, Baden-Württemberg, Germany

## Abstract

Although resource orientation, as a part of health promotion, should play a major role in general practice, the anchoring and realization of resource-oriented approaches remain small in Germany. The aim of this study was to analyze what resource orientation means to general practitioners (GPs) and develop strategies as to how this can be facilitated in GP practice. Within a qualitative research approach, 19 semi-structured telephone interviews were recorded, transcribed, and analyzed using qualitative content analysis. Within the interviews, the inclusion of the patients' individual resources is described as core competence of GPs. Supporting the patients' disease coping strategies and self-help were seen as important by GPs. However, perceptions as to which resources are considered to be fundamental ranged widely across the participant group. The results confirm the important role of resource-oriented approaches in general practice. However, a general definition of resource orientation is needed. In addition, working conditions for GPs need to be taken into account to ensure that these contribute to a healthy work-life balance. The need for GP training was identified to improve communication skills. Further integration of GPs in health promotion and communal structures would be beneficial.

## 1. Background

Resource orientation focuses on individual resources (=“inner potentials”) of the patient to maintain and improve personal health. Thereby, it is mainly characterized by the salutogenic perspective stressing on factors that support human health and well-being. In addition, it overlaps or shares common elements with other concepts used in the context of health promotion, and complementary and alternative medicine such as sense of coherence, resilience, patient empowerment, or self-efficacy.

Traditionally, resource orientation has been researched in the psychological and pedagogical context. Increasingly its importance for primary care is recognized, in particular with regard to the management of chronically ill patients [1–3]. In Germany, in 2002, a pilot project explored the salutogenic orientation in primary care which is a part of resource orientation and described three levels of salutogenic orientation [4, 5]. However, ten years later evidence about the use of resource orientation in general practice is still scarce. 

Nevertheless, resource-oriented approaches are increasingly important for general practitioners (GPs). For the evaluation of a patient's individual resources, GPs work within an ideal setting to discover and promote the resources of the patient based on their long-lasting relationship with the patient. GPs do not only have medical knowledge regarding diseases and therapies but also build a therapeutic relationship with patients over time. This enables GPs to gain knowledge about the patient's coping mechanisms, his or her individual resources and additional available resources in the community settings. GPs can potentially anticipate possible causes of pathogenesis from a somatic, psychosomatic, social, and/or human dimension [6]. Furthermore, GPs are able to assess possibilities for individual approaches to resource orientation based on their insights about the individual patient. This is of special importance considering the rapid increase in the prevalence of chronic diseases and multimorbidity. Commonly and characteristically for the primary care setting there arises a long-standing, close relationship between GPs and their patients. Particularly the continuity of care that is concomitant with this long term relationship is associated with greater satisfaction and better health outcomes [7].

Community resources, such as social services, self-help groups but also sports clubs, kindergartens, or schools are essential to facilitate the identification and the leverage effect of individual resources. However, including community resources in a resource-oriented approach requires a well-established cooperation between doctors and local services [8]. To date, little is known about how GPs are integrated into communities and how this could be optimized within future health care models. 

The study is concerned with the position of resource orientation and resource-oriented approaches in GP practice, using a qualitative research approach and focusing on the treatment of chronically ill patients in primary care. The primary aim was to gain a solid understanding of the meaning of resource orientation from the GP perspective. A further aim was to identify possibilities and specific measures to facilitate resource orientation in the primary care setting.

## 2. Methods

To get a realistic, detailed insight into the individual, personal perception of GPs regarding resource-oriented approaches, a qualitative study design consisting of semi-structured one-to-one interviews was chosen and conducted by telephone [9]. The semi-structured interview guide was developed and refined by conducting two pilot interviews.

GPs were recruited via the quality circle “health promotion practices” that took place from 2002 to 2005 (12 invitations) and via a German General Practice E-mail List server (300 invitations). Inclusion criterion was the contact with resource-oriented approaches. We consecutively included all GPs meeting the inclusion criterion and with interest to take part in the study. 

Individual appointments for the interviews were arranged. All interviews were carried out in the Department of General Practice and Health Services Research, University Hospital of Heidelberg, Germany, and were conducted by Franziska Prüfer. Each interview was recorded digitally and transcribed verbatim. Anonymity was preserved during data analysis, and participants consented on this understanding. The interviews were based on the following questions:

- What does resource-orientation mean to you?
- To what extent are resource-oriented approaches important?
- How can resource-oriented approaches be facilitated in primary care?

The goal of the interviews was explained prior to commencement to prevent misunderstanding and to allow time for answering possible questions that participants may have had. The term “resource orientation” as understood by the researcher was not explained in prior to every interview, in order to prevent bias as to the meaning of resource orientation in the responses. The study was approved by the Ethics Committee of the University of Heidelberg Medical Faculty (S-154/2011).

The interviews took place from May 2011 to August 2011. Each interview lasted between 20 and 30 minutes. The analysis was carried out according to the principles of Mayring's qualitative content analysis [10] and was conducted with the software Atlas.ti. The text material was coded in a deductive and an inductive way. Working independently, Franziska Prüfer and Antje Miksch conducted the primary coding. The codes were clearly defined and linked with representative examples from the original text. The codes were grouped into main and subcategories leading to a categorical system which was discussed in an iterative process among Franziska Prüfer, Antje Miksch, and Stefanie Joos. In case of discrepancies, discussion took place until a consensus was found. The quotations cited were translated from German by Franziska Prüfer and cross-checked by Antje Miksch.

## 3. Results

Altogether, 19 GPs were recruited for the study. Age ranged from 45 to 67 years (mean 55,7 years). Occupational experience as GP ranged between 1 and 37 years. Eighteen were medical specialists in general practice; one was a medical specialist in physical and rehabilitative medicine (Table 1).

Two main categories emerged from this analysis: the meaning (abstract ideas) and the facilitation (concrete ideas) of resource orientation in general practice.  Table 2 illustrates categories, subcategories, and codes.

### 3.1. Meaning

The interviews showed a very heterogeneous understanding of resource orientation which will be illustrated subsequently. Although the foundational understanding appeared similar, GPs focused on different aspects. For the GPs, resource orientation also meant orientating themselves towards their own resources. 

#### 3.1.1. Meaning for the GP

During the interviews, resource orientation was described as a core competence of GPs. For example, “because these [resource-oriented approaches] are the basics of our [GPs'] actions” (GP 10). Most GPs stated that these approaches were partly carried out intuitively. Moreover, resource orientation was considered to be an attitude that focuses on the inner potentials of every individual. Some of the GPs expressed a strong need for a common definition to further establish resource orientation and make it comprehensible for everybody. During the interviews, resources were amongst other things defined “as sources of a healthy development” (GP 16). Resources represented an inner potential that differs quite individually from one person to another. In addition, assistance towards self-help and coping with illness were also considered to be an integral part of resource orientation by the GPs.

*“That after all is our aim, to make people healthy. Healthy not in a sense that everything is still working, but that one can also be satisfied with life and the circumstances within which one is living (…).” (GP 10)*

In addition, the interviews showed the importance of establishing awareness regarding resources. According to the GPs, this is a necessary prerequisite to implement and foster resource-oriented approaches. 

Furthermore, GPs attach importance to resource orientation also with regard to their own resources for preserving and promoting their own health. Most of the GPs were satisfied with their own living and working situation. However, some of the GPs reported periods of exhaustion or burnout. During the interviews, the desire for a good work-life balance was evident. Satisfactory working conditions were emphasized as a core factor in achieving a healthy work-life balance. An appropriate workload, the possibility to exchange ideas with colleagues, and job satisfaction all contribute to the strengthening of GPs' resources.

*“…that I am trying to reduce my employment duties to a bearable extent, so that it is not too much for me and I can really have an open ear for the patients.” (GP 02)*

In addition, acceptance by the GP that the patient declines the GP's preferred therapy method also contributes to the effective resource orientation of GPs.

Participant GPs utilized leisure activities to reduce stress. Music, philosophy, and relaxation methods as well as family and friends were mentioned frequently. A healthy diet, physical activity, and weight loss were further factors mentioned in the context of maintaining a healthy lifestyle.

#### 3.1.2. Supposed Benefits for Patients

The interviews showed the importance of creating a heightened awareness regarding resources. For example,

*“(…) in such a way that a point of view evolves that does not focus on illness, but on a person who is now in this special situation and who with his or her own possibilities (…) will have to cope alone again later on and have to take direct responsibility.” (GP 19)*

#### 3.1.3. Opportunities

For the GPs resource orientation opens the potential for working with chronically ill people to become more satisfying. Interviewees had the impression that patients can increase personal autonomy with the acceptance of direct responsibility for their health and well-being. This can have benefits for both patients and GPs. Patients can improve their satisfaction and awareness of health and in sharing responsibility, GPs can also achieve improved work satisfaction.

*“Well, I believe that patients can profit immensely if one proceeds in this way. It is a much more relaxed working atmosphere for the patient as well. (…) And in return I do not carry the entire responsibility all by myself as a doctor, which is of course a burden in my profession, but can hand over part of it.” (GP 19)*

Additionally, the interviewees explained that when the self-responsibility for health in a patient increases, medical interventions and medication intake can be reduced. As an additional gain, deleterious effects, complications, and costs can be lowered. 

### 3.2. Facilitation

The interviewed GPs did not have a standardized method to stimulate their patients' resources. Methods were very individual and differed from doctor to doctor. In addition, the patient contributed decisively to the choice of a suitable method of facilitation, as not every patient is receptive to the same treatment. Furthermore, the opinions about which resources are most important differed considerably among GPs.

#### 3.2.1. Communication

According to the interviewees, communication is the decisive mean for the facilitation of resource-oriented approaches.

*“For the time being, I would look at it from the point of view that it is essential to enter into a dialog with the patient. This means it cannot be a one-way street, we will have to reach an agreement with each other.” (GP 03)*

In order to conduct an effective doctor-patient conversation, the interviewees considered a common language to be essential. With respect to this, they pointed out that language barriers may emerge during conversations with patients with a migrant background. Additionally, the importance of using a language that is comprehensible to patients was stressed several times. In the context of relevant methods of communication, the interviewees emphasized that questions asked by doctors should be open, for instance, questions about the patients' subjective theories about their illness: “*How do you explain your illness?*” (GP 12) or to strengthen the patient's own competence: “*What helps you? What does not*?” (GP 16). This has two advantages: on the one hand, the patient can become active and reflect on his current situation. On the other hand, the answers given enable the doctor to respond to the patient in an improved way and to look at him or her as a whole.

In addition, the interviews showed how rarely questions are actually asked about a patient's background in a GP's practice. One of the doctors reported that he had only started doing this within the context of resource orientation.

In addition to the asking of questions, reporting about other patients was identified as a possible method for opening communication channels. Examples of patients in similar situations could help to illustrate the presentation of potential effects of therapy. For example:

*“There are certain patients in which it proves to be fruitful, but often only if a specific example is given, of the place where it went wrong.” (GP 08)*

#### 3.2.2. Patient Orientation

Further approaches to put resource orientation into practices were seen in the field of patient orientation, in which it is especially crucial to look at a patient in his entirety. This means that the patient should not be perceived as an object, but as an individual. In the interviews, it was emphasized that the initial task of a doctor is listening to the patient very closely and accepting him in his entirety. For example:

*“(…) that you try to acknowledge the person sitting in front of you as a whole and, above all, that you also work on a relationship-level, that you establish a relationship with the patient by taking him seriously when he comes in.” (GP 06)*

Doing this promotes the creation of a confidential doctor-patient relationship, which enables the cooperation of doctor and patient on the same level. They can then jointly define a resource-oriented treatment based on the individual patient's quality of life. For example;

*“That the whole thing happens inside of a single person, inside of an individual, a personality and that it is always about detecting why this single person, this personality or specifically this patient is sitting in front of me in my practice, the special feature, the unique, the unusual, about where this person stands in the continuum between health and illness (…).” (GP 03)*

#### 3.2.3. Complementary and Alternative Medicine

According to the respondents specific methods of complementary and alternative medicine offer the opportunity to facilitate resource orientation; explicitly mentioned were Feldenkrais, acoustic bowl therapy, magnetic fields, homeopathy, and kinesiology.

*“By this a lot could be adjusted, for example, with acoustic bowls and things could be set in motion beyond curing effects of course. It is both, preventive and curative and it just gives impetus.” (GP 05)*

*“So my own resources I have to build up, retain and improve as well. And therefore I use things as mentioned before: Feldenkrais, acoustic bowl or magnetic fields.” (GP 05)*

Furthermore kinesiology was mentioned as a possibility for the facilitation of resource orientation within primary care.

*“… this I do as mentioned before with half an hour of kinesiology offered in my practice.” (HA 07)*

#### 3.2.4. Lifestyle Changes

Changes of lifestyle can also support the strengthening of patients' healthcare resources, in the view of participating GPs. First and foremost, those lifestyle changes included physical exercise, a healthy diet, and weight loss.

*“…that it is important to make healthy provisions by making it clear to people that they have to eat a healthy diet, exercise and lose excess weight.” (GP 08)*

It was possible to set goals in order to counteract patients' difficulties in putting plans such as regular exercise into practice. Setting realistic goals could also strengthen a patient's motivation.

#### 3.2.5. Settings

The setting (social entities, e.g., schools, districts, and migrant gatherings) was seen as a further possibility to facilitate resource-oriented approaches throughout the interviews. Doctors especially stressed the integration into local structures which was described as a very positive experience:

*“Well, I am a sponsor of a race. Its' first prize goes to the school that enrolls the most students for the run. (…) By handling it that way, you also signal that you think it makes sense to let children exercise without it being about who is the fastest runner but instead about the run as a shared experience.” (GP 06)*

Networking between nonmedical and medical professions was perceived as another starting point for the facilitation of resource orientation. According to the GPs interviewed, networking between professions could create a concept for society as a whole which includes doctors and stimulates resources in many fields.

*“Networking. Actually you would have to network much more in order for this approach to be of more use.” (GP 14)*

Primarily, the settings of school and kindergarten seemed to have the potential to increase the usage of resources. It was particularly emphasized that children should be exposed to principles of health promotion as early as possible. This could either prevent the future development of a disease or at least delay onset. The early development of self-esteem also was seen to be of great importance.

*“It does not matter whether children do sports or play an instrument but it is important that there is something they can relate to which increases their self-confidence as well as their self-esteem. I think that this results in clearly lower rates of drug users.” (GP 11)*

The role of workplace health promotion in preventing burnout and experiences of excessive demand was pointed out as well. From the participating GPs' perspectives, the development of resource-oriented workplaces in collaboration with occupational physicians was an important task, especially considering our aging society. For instance, it would be possible to grant additional breaks to older employees and to consider the employees' age when assigning tasks.

#### 3.2.6. Improvements

A majority of respondents consider the possibility to spend more time with the patients as well as financial reimbursement for consultation as a foundation for the improvement of resource orientation. Furthermore, the integration of resource orientation in GP care by, for example, establishing resource-oriented consultations was discussed. During the interviews, the question which role a GP plays in regard to resource orientation and health promotion and whether this is part of his or her professional role at all was frequently asked.

*“…and where it is retorted whether this even is a doctor's duty.” (GP 14)*

Additionally, respondents expressed their wish for better representation of resource orientation in medical education and research. An emphasis was placed on the necessity of offering patient-oriented counselling techniques.

## 4. Discussion

Within this qualitative interview study the importance of resource orientation for primary care was clearly emphasized by GPs; nevertheless there was a heterogeneous understanding of what resource orientation means. Resource orientation is regarded to be essential for the doctor-patient communication and relationship. Furthermore, the interviews demonstrated that resource orientation plays an important role in a double sense: for the GP's own resources and for the care of patients. In many of our results the close relation between resource orientation and the concept of health promotion became apparent. In contrast to simple resource orientation, this concept concerns all societal elements and consequently an analysis and subsequent strengthening of health resources is in order. A salutogenic perspective is characteristic of both, resource orientation and health promotion.

The interviewees noted that when GPs use resource orientation they do not merely see the patient as a person with an illness, but rather as a subject that is regarded in his or her entirety. Since medical measures are taken based on patient's individual resources, most GPs see the consideration of individual aspects as a core area within primary care, similar to the GPs within this study. In 2011, the European association WONCA defined six core competences which are required from a GP, which include an individualistic treatment and a holistic model [11]. In this context a holistic treatment means the embeddedness in the societal environment. As individualistic and holistic treatments are crucial in resource orientation, it can be suggested that resource-oriented acting actually is a fundamental aspect in the working routine of GPs, even if this is not represented by primary care research. Previous studies have shown that GPs feel that they have insufficient capacities to execute health promotion on a daily basis [12]. However, this study shows that resource-oriented methods are already being used intuitively and therefore could be considered as fundamental approaches within primary care that should be addressed more anticipatory. Furthermore, the fact that resource orientation is one of a GPs core competences should be given a larger focus in the discussion of the redefinition of tasks and responsibilities in the field of health promotion. 

The results indicate that resource orientation plays a crucial role in relation to GPs' own health. Despite the fact that burnout syndrome or exhaustion were only rarely mentioned in interviews, it is now widely known that doctors are paradoxically one of the occupational groups most at risk of health impairment. Due to the fact that a doctor's state of health may also impact on a patient's medical care and thereby a patient's health, it is clearly necessary to promote health and well-being among medical specialists. Despite the high percentage of males in this study, the compatibility of family and career becomes increasingly important given the rising number of women in medicine [13, 14]. A vital aspect in order to increase doctor's well-being is a change in working conditions. A more in-depth examination of methods to increase resilience and minimise work-related stress would also be beneficial. Moreover, the results pointed out that a contrast to work, for example, leisure activities or relaxation techniques were an important resource in order to promote medical specialists' general well-being. In order to combat overwork, doctors should further learn when to say no and to distance themselves [15]. Furthermore, psychological problems shouldn't be seen as taboo anymore, in order for doctors to be able to admit sick periods and cater to their own needs. Other studies concerning stress in the medical profession report that doctors need to exchange ideas on stressful situations in a community, which would be easy to implement, for example, by utilising supervision [16].

In addition to an increased awareness of their own resources, doctors may also feel relief if patients accept more self-responsibility for their health. In order to do so, resource consciousness as well as watchfulness of one's own body needs to be facilitated among patients. They may, for instance, become more autonomous, active and assume more responsibility by receiving instructions from their medical practitioner. Thereby, patients can prevent and counteract mild diseases without straining their own health. The provision of information and skilled advisers in self-help can promote confident interaction between doctors and patients. Thereby, this method increases patient competence and contributes to shared-decision making [17]. A limitation, however, is that patients are often not motivated enough to be engaged in independently taking responsibility for their own health [18], especially concerning risk factors that cannot directly be sensed, for example, high blood pressure. Lifestyle changes are often not considered necessary by patients. In this way, the patient should learn how to cope with his or her illness with the help of the GP. Therefore patients need to be counseled and supported by their GP. This is especially true for methods of complementary and alternative medicine which are according to this study an important source of resource orientation. This is in agreement with previous studies for the field of herbal medicine showing that herbal medicine meets the patients' needs for autonomy, support, and self-care [18]. 

In order to be able to implement resource orientation more effectively, doctors were asked for a general definition of the term. Bringing together the existing approaches around resource orientation with the aim to establish a general definition would be an important step for patient care but also for education and further research.

The results also indicate that currently there is no structured advancement in implementing resource orientation. In order to improve this, a working communication between a doctor and his patient is necessary, due to the fact that this exchange can promote compliance as well as significantly increase the exchange of information [19]. Techniques which could possibly foster patient-oriented care could particularly include asking questions as well as well-known communication techniques such as active listening. Recent research shows that balancing dialogues are a further method of communication, which can alleviate the structuring of long-term GP treatment of chronically diseased patients. In balancing dialogues, a joint treatment is evaluated, and suggestions of improvement are discussed [20].

The results also show that teaching communication competence as well as resource-oriented approaches are essential in medical training and further education. Existing competency-based curricula such as the CanMeds are particularly recommended as a guiding manual for undergraduate and postgraduate education. Within the CanMeds concept health promotion and resource-oriented approaches are described in the role of the “Health advocate” [21]. Integration into undergraduate and postgraduate curricula is essential to make medical students sufficiently sensitive and strengthen their personal skills in these topics. A manual, which gives practical consultation advice for the use of resources, would be a critical prerequisite in order to implement and promote resource orientation. However, this requires a common definition and a stronger evidence base for resource orientation. 

With regard to facilitation communal resources can offer great potential to treat patients in a resource-oriented manner. Some doctors questioned were already integrated in community activities. However, they reported that integration into communities is largely dependent upon the doctor's own commitment due to the fact that structures, which could facilitate integration, are generally lacking. In order to improve these conditions a better network of all actors involved is a desirable solution. This should not only include all health-related professions (doctors, nurses, and allied health care professionals), but also other professional groups within the community, for example, in the fields of education and employment. A key element in supplying healthcare for chronically ill patients is an integration of networking as described within the chronic care model, an approach which has currently also been discussed [8, 21]. Communal health conferences were introduced for example in one federal state of Germany in order to supply for network structure. These serve as a platform for communication and coordination for all actors in health care, and aim to create networks on relevant topics, in order to jointly solve local problems [22]. Additionally, further models for successful interprofessional collaboration should be advertised and promoted.

In the interviews, the role of GPs was also discussed, and it was mentioned that individual health care is a GP's primary duty within health promotion. Doctors can introduce patients to health promotion offers, which already exist within the community, or can alternatively offer these in their own practices (e.g., nutrition consultation). Often however, doctors lack knowledge of health care offers already established within their communities. Another approach in further incorporating GPs into the community and fostering their work relief is a possible restructure of ambulant health care by strongly integrating other health care professionals. After all, most patients visiting a GP already show symptoms. Resource orientation should, however, also be applied to healthy individuals.

The results, moreover, suggested that there are further possibilities to accommodate for resource-oriented approaches in health care. A resource-oriented consultation for chronically ill patients could increase the focus on healthy and positive aspects in the patient's life and improve his quality of life. An integration of resource orientation in existing structures, for example, established medical check-ups also seems promising. Lastly, the use of a resource-oriented questionnaire, which concerns the patients' different areas of life as well as their capacities, could prove reasonable. 

Our study has several limitations. One could, for instance, view the inclusion criterion as a limitation, “experience in resource orientation”, for example, was not further defined. An imbalance of gender representation may also be understood as a limitation. However, our aim was not to draw conclusions on a representative basis, but to explore the respective GPs' personal insights on resource orientation. The remote interview method has some limitations regarding the creation of an atmosphere of trust. However, this approach was chosen purposefully to be able to include GPs from around Germany, with an awareness of the bias that can arise from this method [23]. A further limitation could be that no definition of resource orientation was given to the participants before the interviews. This method was chosen in order to prevent bias regarding the meaning of the words “resource orientation.” Furthermore, the interviewees were not asked to what extent they implement resource-oriented methods, but rather how they would do so. This study does only consider GPs, which seems to give a limited view of the subject. To broaden and enhance the results of this study, the next step would be to interview patients as well as other health professionals and to analyze the generated hypotheses in further quantitative studies.

## 5. Conclusion

Resource orientation, as a part of health promotion, plays an important role in GPs' daily work. Therefore it should be increasingly taken into account. Furthermore, resource orientation should be focused in medical curricula as well as in daily work routine of GPs and other health professionals. Resource-oriented skills could, for example, be taught in communication classes or quality circles. Moreover, GPs should be more integrated in communal structures. With regard to the demographic development this can contribute to respond the growing demand of health care and the increasing workload and burden within primary care. Additionally, the duty of the patients is to become aware of their resources. They need to take over direct responsibility for their health and health related problems which could lead to a better quality of life and self-efficacy of patients especially those with chronic conditions. In this way, resource orientation could lead to a reduced demand of medical services and, thus, to a reduction of GPs' workload.

## Figures and Tables

**Table 1:** Sociodemographic characteristics of the study sample (*n* = 18*).

Gender	
Female	2
Male	16
Age in years (mean, range)	55.7 (45–67)
Working experience in general practice in years (mean, range)	20.4 (1–37)
Type of practice	
Sole practitioner	6
Group practice	10
Location of practice	
City	5
Small town	4
Rural area	7

*Sociodemographic data was available for 18 respondents only.

**Table 2:** GPs' opinions regarding the meaning and facilitation of resource orientation.

Main category	Subcategory	Code
Meaning Definition: abstract ideas about the meaning of resource orientation by GPs	Meaning for the GP	Core competence Attitude Definition of resources Help towards self-help Help for disease coping Awareness of patients' resources Awareness of own resources
	Supposed benefits for patients	Accepting direct responsibility
	Opportunities	Increased job satisfaction Increased autonomy Increased satisfaction of the patient Less side effects Cost reduction

Facilitation Definition: specific ideas and methods of implication of resource-oriented approaches in GP practices and social environments	Communication	Patient-doctor conversation Asking questions Usage of case studies
	Patient orientation	Holistic approach Individuality of the patient Doctor-patient relationship Interaction Doctor accepts the patient Basis of trust Listening Patient satisfaction
	Complementary and alternative medicine	Feldenkrais Homeopathy Psychotherapy Acoustic bowl therapy Kinesiology Anthroposophy
	Lifestyle changes	Target agreement Physical activity Healthy diet Weight loss
	Settings	Communal structures Networking Promotion during childhood Kindergarten School Workplace health promotion
	Improvements	Health care system Training Research
